# The Past, Present, and Future of Public Health Surveillance

**DOI:** 10.6064/2012/875253

**Published:** 2012-08-05

**Authors:** Bernard C. K. Choi

**Affiliations:** ^1^Injury Prevention Research Centre, Medical College of Shantou University, Shantou 515041, China; ^2^Department of Epidemiology and Community Medicine, University of Ottawa, Ottawa, ON, Canada K1H 8M5

## Abstract

This paper provides a review of the past, present, and future of public health surveillance—the ongoing systematic collection, analysis, interpretation, and dissemination of health data for the planning, implementation, and evaluation of public health action. Public health surveillance dates back to the first recorded epidemic in 3180 B.C. in Egypt. Hippocrates (460 B.C.–370 B.C.) coined the terms endemic and epidemic, John Graunt (1620–1674) introduced systematic data analysis, Samuel Pepys (1633–1703) started epidemic field investigation, William Farr (1807–1883) founded the modern concept of surveillance, John Snow (1813–1858) linked data to intervention, and Alexander Langmuir (1910–1993) gave the first comprehensive definition of surveillance. Current theories, principles, and practice of public health surveillance are summarized. A number of surveillance dichotomies, such as epidemiologic surveillance versus public health surveillance, are described. Some future scenarios are presented, while current activities that can affect the future are summarized: exploring new frontiers; enhancing computer technology; improving epidemic investigations; improving data collection, analysis, dissemination, and use; building on lessons from the past; building capacity; enhancing global surveillance. It is concluded that learning from the past, reflecting on the present, and planning for the future can further enhance public health surveillance.

## 1. Introduction

The term “surveillance”, derived from the French roots, *sur* (over) and *veiller* (to watch) [1], is defined in the dictionary as the “close and continuous observation of one or more persons for the purpose of direction, supervision, or control” [2]. For the purpose of this paper, the following definition is used, “*Public health surveillance is the ongoing systematic collection, analysis, interpretation and dissemination of health data for the planning, implementation and evaluation of public health action*” (see Section 2.3 below).

Public health surveillance is considered to be an essential public health function [3, 4]. A public health system is said to have five essential functions: population health assessment, health surveillance, health promotion, disease and injury prevention, and health protection [5]. Public health surveillance is considered the best weapon to avert epidemics [6]. 

The objective of this paper is to provide a review of the past, present, and future of public health surveillance. The section on the past includes an account of major epidemics in human history, the historical milestones in the development of public health surveillance, and the historical evolvement of the concepts and definitions of public health surveillance. As much as possible, original historical documents have been consulted and quoted in this paper. The section on the present describes the uses and components of public health surveillance as we know it today. The section on the future reviews the literature concerning possible scenarios and proposed directions by various authors for future development of public health surveillance.

## 2. The Past

### 2.1. Records of Major Epidemics in Human History

Public health surveillance dates back to the time of Pharaoh Mempses in the First Dynasty, when an epidemic was first recorded in human history [7]. Manetho, the Egyptian priest and historian, stated in his list of pharaohs, “*Mempses, for eighteen years. In his reign many portents and a great pestilence occurred*” [8, 9]. The “great pestilence” is now known to have occurred in 3180 B.C. (Table 1). Table 1 provides a list of major epidemics recorded in history. It also provides the necessary background and context for the discussion below of the major milestones and historical development of the concepts and definitions of public health surveillance. 

According to Marks and Beatty, the three most devastating epidemics to hit the human race were “The Plague of Justinian” (A.D. 541–591) which lasted 50 years, "The Black Death" (1348–1351) which lasted 4 years, and “Spanish Influenza” (1918) which lasted five months [9] (Table 1). From an analysis of Table 1, it can be seen that three types of information were included in the historical records of epidemics. These are health outcomes, risk factors, and interventions (Table 2). These are also the types of information that should be included in a modern day public health surveillance system. They are the forces guiding the changes in public health. Health outcomes measure the state of public health. Risk factors move the state of public health towards undesirable health outcomes, and interventions if successful move the state of public health towards desirable health outcomes.

### 2.2. Major Milestones in the Historical Development of Public Health Surveillance

Simply recording epidemics is not exactly public health surveillance as we know it today. Major milestones in the historical development of concepts in public health surveillance are given in Table 3. The first record of an epidemic was made in 3180 B.C., starting the practice of collecting and recording data.

The idea of collecting and analyzing data dates back to Hippocrates (460 B.C.–370 B.C.) [31], an ancient Greek physician who is also known as the father of medicine and the first epidemiologist [40, 41]. He is credited with being the first person to believe that diseases were caused naturally and not because of superstition and gods [42]. Disease was a consequence of local conditions, which had to be favourable for a particular disease to occur. He introduced the concept of categorizing illnesses as acute (short duration) or chronic (long lasting). He also coined the terms endemic (for diseases usually found in some places but not in others; steady state) and epidemic (for diseases that are seen at some times but not others; abrupt change in incidence) [31, 43]. In his book *On Airs, Waters, and Places* he wrote, “*The men are subject to attacks of dysentery, diarrhea, hepialus, chronic fevers in winter, of epinyctis, frequently, and of hemorrhoids about the anus. Pleurisies, peripneumonies, ardent fevers, and whatever diseases are reckoned acute, do not often occur, for such diseases are not apt to prevail where the bowels are loose. Ophthalmies occur of a humid character, but not of a serious nature, and of short duration, unless they attack epidemically from the change of the seasons. And when they pass their fiftieth year, defluxions supervening from the brain, render them paralytic when exposed suddenly to strokes of the sun, or to cold. These diseases are endemic to them, and, moreover, if any epidemic disease connected with the change of the seasons, prevail, they are also liable to it.”* [44]. According to the Hippocratic definition, an endemic is a disease determined by the nature of a certain place, and climatic, hydrological, and behavioural determinants are seen as the main forces [45]. This provides the concept of collecting data on place, natural environment and people for determination of illness.

The first public health action that can be attributed to surveillance occurred during the 1348 bubonic plague epidemic which started the “Black Death”. The Venetian Republic appointed 3 guardians of public health to detect and exclude ships which had infected people aboard [26, 27]. Quarantine as a means to control the spread of infectious diseases was used again in 1377 in Marseilles to detain travellers from plague-infected areas for 40 days [12].

The concept of systematic ongoing collection of mortality data was first used in 1532 when the town council of London, England started to keep a count of the number of persons dying from the plague [28]. These Bills of Mortality were collected on and off for over 100 years [46]. However, these data were not used for surveillance purpose until the 1600s, when the clerks of London reported the number of burials and causes of death to the Hall of the Parish Clerk's Company and released in a weekly Bill of Mortality [33]. 

Comprehensive analysis and interpretation was introduced by John Graunt (1620–1674), a haberdasher and serious amateur scientist in London, who analyzed the weekly bills and published in 1662 his book *Natural and Political Observations Made upon the Bills of Mortality* [29]. For this work he was subsequently elected a fellow of the Royal Society, whose members initially were uncomfortable with the idea of a haberdasher being elected [47]. Graunt was the first to quantify the patterns of disease and to understand that numerical data on a population could be used to study the cause of disease [31]. He was the first to estimate the population of London and to count the number of deaths from specific causes. 

The practice of epidemic field investigation began with the personal diary Samuel Pepys (1633–1703) kept from 1660 until 1669. His diary is an important primary source of data and first-hand account for London, with personal revelation and eyewitness reports of many great events [48]. During the “Great Plague of London” in 1665, Pepys' diary made almost daily reference to the epidemic, “*15th [June]… The towne grows very sickly, and people to be afeard (afraid) of it: there dying this last week of the plague 112, from 43 the week before… 20th [July]…Walked to Redriffe, where I hear the sickness is, and indeed is scattered almost every where, there dying 1089 of the plague this week… 31st [August]… In the City died this week 7496, and of them 6102 of the plague… 30th [November]… Great joy we have this week in the weekly Bill, it being come to 544 in all, and but 333 of the plague*” [49]. Not using the modern terminology, he actually introduced the concept of proportionate mortality, or the proportion of total deaths resulting from the index disease [50]. According to the numbers kept in Pepys' diary, the proportionate mortality for plague was 81% (6102/7496) on August 31, 1665, which decreased to 61% (333/544) on November 30, 1665 when the epidemic started to subside (Table 4). These numbers recorded by Pepys from the beginning of June to the end of November indicate the effectiveness of the natural intervention, that is, the coming of the November frosts and the winter [7]. The plague ended with the “Great Fire of London” in 1666 that destroyed and cleansed the overcrowded neighbourhoods [9].

Legislation for surveillance was first introduced in 1741 in the Americas, when the colony in Rhode Island passed an act requiring tavern keepers to report contagious disease among their patrons. In 1743, the colony passed a law requiring the reporting of smallpox, yellow fever, and cholera [30]. This started the concept of compulsory reporting of infectious diseases.

Surveillance was felt to need to link to policy development. In 1776, Johann Peter Frank in Germany advocated a comprehensive form of public health surveillance which dealt with school health, injury prevention, maternal and child health, and public water and sewage treatment [30]. Frank formulated comprehensive health policy which had considerable impact both within Germany and in countries such as Hungary, Italy, Denmark, and Russia that had close cultural contact with Germany [51].

In addition, leaders of the French revolution (1788–1799) declared that the health of the people was the responsibility of the state [27]. This started the concept of a welfare state.

Surveillance efforts were used to develop legislation and social change. Sir Edwin Chadwick, secretary of the Poor Law Commission in England, using surveillance data, demonstrated the link between poverty and disease [31]. He published the report of 1834 recommending the reform of the old Poor Law. The new Poor Law system was in existence until the emergence of the modern welfare state after the Second World War (1939–1945) [52]. The New Poor Law is considered to be one of the most “far-reaching pieces of legislation of the entire Nineteenth Century” [53]. At about the same time, Louis-René Villermé (1782–1863) studied the mortality rate variations across the 12 *arrondissements* (districts) of Paris 1817–1826, by district, population density, and income and showed the association between poverty and mortality [54]. 

William Farr (1807–1883) is recognized as the founder of the modern concept of surveillance [32]. In 1836, the General Register Office was established in England and Wales to provide more accurate and complete mortality data [25]. Medical certification of death and universal death registration was introduced in 1837 [55]. Farr was the first Compiler of Abstract (medical statistician) at the General Register Office. He began the practice of collecting and analyzing vital statistics to describe the impact of diseases in various populations [32]. From 1838 to 1879 (for 41 years), he concentrated his efforts on collecting vital statistics, on assembling and evaluating those data, and on reporting his results to both the responsible authorities and to the general public [56] and created a modern surveillance system [55]. 

Surveillance was proposed to link to statewide public health infrastructure. In the United States, Lemuel Shattuck published in 1850 his “Report of the Massachusetts Sanitary Commission”, based on the survey of sanitary conditions in Massachusetts [31]. This report was a landmark publication that related death, infant and maternal mortality, and communicable diseases to living conditions [56]. In this report, Shattuck proposed the creation of a permanent statewide public health infrastructure and recommended establishing health offices at the state and local levels in order to gather statistical information on public health conditions [57]. He recommended a decennial census, standardization of nomenclature for diseases and causes of death, and the collection of health data by age, sex, occupation, socioeconomic level, and locality [31]. Although the legislature did not adopt his comprehensive plan, his specific proposals became routine public health activities over the course of the twentieth century.

John Snow (1813–1858), an anaesthesiologist, is famous for his investigations into the causes of the 19th century cholera epidemics and is also known as the father of modern epidemiology [33, 58]. In 1849, Snow mapped cholera cases in London and identified the source of the outbreak as the public water pump on Broad Street (now Broadwick Street). Using a dot map, he illustrated the cluster of cholera cases around the pump. Snow wrote: “*On proceeding to the spot, I found that nearly all the deaths had taken place within a short distance of the [Broad Street] pump. There were only ten deaths in houses situated decidedly nearer to another street-pump. In five of these cases the families of the deceased persons informed me that they always sent to the pump in Broad Street, as they preferred the water to that of the pumps which were nearer. In three other cases, the deceased were children who went to school near the pump in Broad Street… With regard to the deaths occurring in the locality belonging to the pump, there were 61 instances in which I was informed that the deceased persons used to drink the pump water from Broad Street, either constantly or occasionally… The result of the inquiry, then, is, that there has been no particular outbreak or prevalence of cholera in this part of London except among the persons who were in the habit of drinking the water of the above-mentioned pump well. I had an interview with the Board of Guardians of St James's parish, on the evening of Thursday, 7th September, and represented the above circumstances to them. In consequence of what I said, the handle of the pump was removed on the following day*” [59]. On September 8, 1854, Snow removed the pump handle and the epidemic waned [13, 60]. Snow's work is a good illustration of collection, analysis, interpretation, and dissemination of data leading to public health intervention.

Systematic reporting of various diseases started in the United States in 1874 in Massachusetts [61]. The Massachusetts State Board of Health inaugurated a plan for weekly voluntary reporting of prevalent diseases by physicians [34]. A sample postcard was designed to “*reduce to the minimum the expenditure of time and trouble incident to the service asked of busy medical men*” [35]. In Europe mandatory reporting of infectious diseases started in Italy in 1888, and in the United Kingdom in 1890. Finally, the 20th century brought the expansion and diversification of public health surveillance systems. Table 3 gives some of the more important events related to the development of surveillance in the last century. 

The United States has been taking a lead in the development of concepts and models for public health surveillance. A detailed account of the development of the public health surveillance system in the United States for 1850–1950 is given elsewhere [30, 36, 62]. It is of interest to know the brief history of the US agency known as “CDC” that is responsible for public health surveillance in the United States. The CDC was founded in 1942 as the Office of National Defense Malaria Control Activities [63, 64]. Atlanta was chosen as the location because malaria was endemic in the Southern US. In 1946, the agency changed its name to Communicable Disease Center, and hence the acronym “CDC” [63]. In 1947, CDC took over the Public Health Service Plague Laboratory in San Francisco, thus acquiring an Epidemiology Division. In 1955, CDC established the Polio Surveillance Program, in order to prove that an epidemic could be traced to a single vaccine manufacturer [33]. In 1961, CDC took over publication of Morbidity and Mortality Weekly (MMWR). The Communicable Disease Center was renamed the Center for Disease Control in 1970, then the Centers for Disease Control effective 1980 [63, 64]. An act of the United States Congress appended the words “and Prevention” to the name effective 1992. However, Congress also specified that the agency continue to use the acronym “CDC” because of its recognition within the public health community and among the public [65]. 

Globally, the public health surveillance program is coordinated by the World Health Organization (WHO). In 1965, the Director General of the World Health Organization established the epidemiological surveillance unit in WHO's Division of Communicable Diseases [33, 38]. The first communicable disease surveillance report was published in 1966. In 1968, the 21st World Health Assembly established surveillance as an essential function of public health practice [39] (Table 3).

### 2.3. Historical Development of the Concepts and Definitions of Public Health Surveillance

Table 5 shows the historical evolvement of the concepts and associated definitions of public health surveillance.

In 1662, John Graunt first suggested in his book *Natural and Political Observations Made upon the Bills of Mortality* the need for ongoing systematic collection of data and proposed the basic principles for data analysis and interpretation, although he did not conceptualize the link of surveillance information to public health practice [29] (Table 5). 

In those days, mortality data collection was simple but routine. Every night, towards twelve o'clock, a cart goes about with a lantern and a bellman (or sexton), and as he rings the bell, he cries out, “Bring out your dead!” As described by Graunt, “*When any one dies, then, either by tolling, or ringing of a Bell, or by bespeaking of a Grave of the Sexton, the same is known to the Searchers, corresponding with the said Sexton. The Searchers hereupon repair to the place where the dead Corps lies, and by view of the same, and by other enquiries, they examine by what Disease or Casualty the Corps died. Hereupon they make their Report to the Parish Clerk, and he, every Tuesday night, carries in an Accompt of all the Burials and Christnings happening that Week, to the Clerk of the Hall. On Wednesday the general Accompt is made up and printed, and on Thursday published and dispersed to the several Families who will pay four Shillings per Annum for them*” [81]. 

Graunt's method of data analysis was to reduce voluminous data to a few perspicuous tables (Table 5). Using this method, he was the first to recognize that there were more male than female deaths in London. He tried to interpret the findings and was able to explain the observation by noticing that there were more males than females by counting the number of births, and he suggested that this phenomenon in London should be searched for elsewhere. In Graunt's words, “*There have been Buried from the year 1628, to the year 1662, exclusivè, 209436 Males, and but 190474 Females: but it will be objected, that in London it may indeed be so, though otherwise elsewhere; because London is the great Stage and Shop of business, wherein the Masculine Sex bears the greatest part. But we Answer, That there have been also Christned within the same time, 139782 Males, and but 130866 Females,… What the Causes hereof are, we shall not trouble our selves to conjecture, as in other Cases, onely we shall desire, that Travellers would enquire whether it be the same in other Countries*” [82].

Graunt's concepts described in 1662 (Table 5) can be translated to a first definition of public health surveillance as follows: surveillance is the successful analysis of population-based ongoing data (such as death records) to reduce volumes of data to a few easy-to-understand tables, then interpret them, and prepare a few brief and precise paragraphs, so as to gain profit from the data analysis, in order to understand the increase and decrease of diseases [7].

A contemporary of Graunt, Sir William Petty, in his 1687 essay on “Mankind and political arithmetic”, termed the science of Graunt “*Political Arithmetic*” [46, 66, 83]. This term is a good description for today's public health surveillance, which requires arithmetic skills for analysis of data and a keen political sense for interpretation of results.

Before 1963, the term surveillance was used initially in public health to describe the close monitoring of persons who, because of an exposure, were at risk for developing highly contagious and virulent infectious diseases [84]. These persons were monitored so that, if they exhibited symptoms of disease, they could be quarantined to prevent spreading the disease to others. 

In his classic 1963 paper, Alexander Langmuir (1910–1993), chief epidemiologist of US CDC, defined surveillance for a disease to mean “*the continued watchfulness over the distribution and trends of incidence through the systematic collection, consolidation, and evaluation of morbidity and mortality reports and other relevant data*” [67]. He illustrated this application with four communicable diseases: malaria, poliomyelitis, influenza, and hepatitis [67]. He explained that the data and their interpretations must be disseminated to all who have contributed and to all others who need to know [33]. But his definition did not include direct responsibility for disease control activities [27]. 

In 1968, the 21st World Health Assembly adopted the concept of population surveillance which was defined as “*the systematic collection and use of epidemiologic information for the planning, implementation, and assessment of disease control*” [69]. The Assembly expanded Langmuir's definition to include the assumption that surveillance information is collected in order to take appropriate action to improve health outcomes [33]. In other words, surveillance is “information for action” [70]. The Assembly also affirmed the three main features of surveillance: (a) the systematic collection of pertinent data, (b) the orderly consolidation and evaluation of these data, and (c) the prompt dissemination of results to those who need to know, particularly those in position to take action [69].

The 1986 CDC definition of surveillance reflects Langmuir's view [67] that the concept of surveillance did not encompass direct responsibility for control activities and avoids the use of the term surveillance for control activities, although it states that the final link in the surveillance chain is the application of these data to prevention and control [71]. 

The 1988 definition by Stephen Thacker and Ruth Berkelman is very similar to the 1986 CDC definition. While the 1986 CDC definition uses the term “*epidemiologic surveillance,*” the 1988 Thacker and Berkelman definition introduces the new term “*public health surveillance.*” Epidemiologic surveillance focuses on using surveillance information for epidemiologic research, while public health surveillance focuses more on public health practice [85, 86]. Thacker and Berkelman compared the distinctions between public health surveillance and epidemiologic research and decided that the term epidemiologic surveillance is misleading, and surveillance does not equal research [30].

In his 1998 paper on “Perspectives on epidemiologic surveillance in the 21st century”, Bernard Choi presents arguments why it is important for epidemiologic surveillance to come back full circle in the 21st century and become once again the focus of health research: “*Epidemiologic surveillance dates back to the time of John Graunt … In the subsequent 300 years, however, the focus of health research shifted to sample-based studies: cross-sectional, cohort and case-control studies, and clinical trials. In recent decades, awareness of the limitations of sample-based epidemiologic studies has grown. … [H]ealth research can be conducted in the next century using well-maintained and well-validated surveillance databases*” [74]. Epidemiologic research studies that are sample-based are subject to errors caused by the “False Positive Research Cycle”: false positive associations (positive associations that are not true) will continue to be confirmed by a multitude of subsequent studies that are designed to test a hot topic due to an initial false positive report that is incorrect (hot topic bias), and subsequent amplification of the errors through cycles caused by the tendency of authors to write up and submit positive findings but not the true negative findings (positive results bias) and of editors to accept and publish positive findings (editor's bias) [74, 87, 88]. One way to resolve these errors is population-based epidemiologic surveillance. Choi's 1998 [74] definition of surveillance stresses on the concept of “*population-based*” (Table 5). Resources required for population-based epidemiologic surveillance systems can be formidable, but progress in technology and informatics may soon make implementation much easier to achieve [89].

More recent definitions of surveillance, including the 2001 US Centers for Disease Control and Prevention (CDC) [75] and 2006 Public Health Agency of Canada (PHAC) [77] definitions, emphasize on “*public health action.*” The World Health Organization has three webpages that provide definitions of surveillance. The definitions are very similar, except that the phrase “*action can be taken*” on one webpage is interpreted as “*the planning, implementation, and evaluation of public health practice*” on the other two webpages (Table 5).

Table 6 shows a comparison of the evolvement of definitions of public health surveillance over time, from 1662 to 2012. It can be seen that while the components “ongoing,” “systematic,” “collection,” “analysis,” “interpretation,” and “dissemination” have been consistent in the definitions, there are changes in the other components. For example, “epidemiologic surveillance” shifts to “public health surveillance”; “mortality data” to “health data,” and “disease control” to “public health action” (Table 6).

Based on an examination of the trend of use of terms and the most popular components of various definitions given in Table 6, the working definition of surveillance for the purpose of this paper is “*Public health surveillance is the ongoing systematic collection, analysis, interpretation and dissemination of health data for the planning, implementation and evaluation of public health action.*”

## 3. The Present

It is useful to provide an overview of the current status of public health surveillance and its basic principles and concepts. 

For further information, interested readers can consult books written on the basic principles of public health surveillance [85, 90–92]. The 2000 book edited by Teutsch and Churchill [91] was considered in a 2001 article in the American Journal of Epidemiology [93] as a wonderful one-stop source of information on public health surveillance. There is one book which specifically addresses global surveillance of behavioural risk factors [94]. There are books on the statistical methods for public health surveillance [95] and public health informatics [96, 97].

### 3.1. Uses of Public Health Surveillance

The World Bank described six categories of uses of public health surveillance [98].

1. Recognize cases or clusters of cases to trigger interventions to prevent transmission or reduce morbidity and mortality.
2. Assess the public health impact of health events or determine and measure trends.
3. Demonstrate the need for public health intervention programs and resources, and allocate resources during public health planning.
4. Monitor effectiveness of prevention and control measures and intervention strategies.
5. Identify high-risk population groups or geographic areas to target interventions and guide analytic studies.
6. Develop hypotheses that lead to analytic studies about risk factors for disease causation, propagation, or progression.

Table 7 shows the above six categories of uses, with examples from various sources to illustrate the categories.

### 3.2. Components of Public Health Surveillance

Public health surveillance starts with defining the type of data to collect (systematic data framework development) [74], and then the public health surveillance process cycles through three stages: data collection, analysis and interpretation, and the timely dissemination of findings [77]. In addition, the surveillance system should be able to evaluate public health actions (including the surveillance system itself which is a public health action). 

A step-by-step guide is available for the creation of a new surveillance system when existing systems cannot answer a specific public health questions or address new information need [100].

#### 3.2.1. Data Framework

The first question when setting up a new surveillance system is what categories of information should be tracked by the surveillance system [101]. The data framework is usually defined in terms of indicators. An indicator is a measurable factor that allows decision makers to estimate objectively the size of a health problem and monitor the processes, the products, or the effects of an intervention on the population [102]. The number of potential indicators for tracking is enormous and must be systematically narrowed down [74]. Three criteria for indicator framework development are sound, practical, and usable [101]. The process of indicator framework development involves several steps: conducting literature review, expert consultation, and Delphi surveys to get a consensus on a list of indicators and evaluating availability and quality of data [74]. Delphi survey is a method that requires experts to answer questionnaires in two or more cycles, with a feedback summary of experts' opinion after each cycle, in order to converge towards a consensus [103].

A conceptual framework for health information, the Health Template, was put forward in 1991 by the National Task Force on Health Information [104]. The Health Template classifies health information into three major areas: individual characteristics, external milieu, and “health-affecting” interventions and can potentially be used as a model for selecting indicators. Among the indicators, the definition of what constitutes a “case” is important, especially in infectious disease surveillance [98]. Case definitions for surveillance purposes may be different from the criteria used for clinical diagnosis [105]. For noncommunicable diseases, the World Health Organization recommends a stepwise approach to surveillance that has a core and expanded set of indicators [70].

#### 3.2.2. Data Collection

After defining the public health decisions that require information (data framework), the data collection stage begins by choosing the best sources and methods for gathering the data that are needed. This may need to balance competing needs for timeliness, simplicity, and completeness. 

Key data collection approaches include the following.

1. Health surveys: examples are surveys of the environmental, behavioural (e.g., smoking and physical activity), and biological risk factors of populations [94].
2. Administrative data: routine administrative data come from many sources [106], including population-based systems (e.g., vital records), provider-based systems (e.g., physician, laboratory, and hospital records), payor systems (e.g., Medicare), and the records of administrative entities set up for other purposes (e.g., the Environmental Protection Agency or judicial sources) [107].
3. Mandatory reports: examples are mandatory reporting of communicable disease cases (e.g., tuberculosis, syphilis, and whooping cough) [108].
4. Voluntary reports: examples are voluntary reports on adverse outcomes due to drugs, vaccines, consumer products, accidents, and vaccine-preventable or notifiable diseases [77].
5. Studies of special groups: examples are detailed studies of selected population subgroups (e.g., people living with HIV and AIDS) and of “cases” with particular characteristics in both human and animal populations (e.g., identifying cases of variant Creutzfeldt Jacob disease in people and bovine spongiform encephalopathy or “Mad Cow Disease” in cattle) [77].

Data sources vary from country to country depending on the stage of development and sophistication of public health services and laboratory facilities [12], and the availability of computers and computer networks [27]. Advantages and disadvantages have been discussed of the following data sources for public health surveillance: death certificates, case reporting, epidemic reporting, laboratory reporting of etiological agents, individual case investigation, epidemic field investigation, infectious disease markers surveys, animal reservoir and vector distribution studies, demographic data, environmental data, hospital records, general practitioners records, public health laboratory reports, disease registries, drug and biologics utilization and sales data, absenteeism from school or work, health and general population surveys, and newspaper and news broadcasting reports [27].

For noncommunicable diseases, the World Health Organization's stepwise approach to surveillance collects data through questionnaire, physical measurements, and biochemical measurements [70].

#### 3.2.3. Data Analysis/Interpretation

The analysis and interpretation stage includes expert analysis of the data that have been collected to determine the occurrence of a health concern and the characteristics and behaviours of people with a health concern as well as changes over time. Surveillance data initially should be analysed in terms of time, place, and person [109, 110], by looking at time trends and geographic distribution and comparing age, sex, and population groups [77]. 

More advanced data analysis for surveillance data are available [111]. Examples include space-time clustering [112], time-series analysis [113, 114], geospatial analysis [115], life tables [116], logistic regression [117], trend and small area analysis [77], mathematical models to study the dynamics of infection within communities of people [118, 119], and methods for the forecast of epidemics based on surveillance data [120–122].

Data analysis must be followed by interpretation. Interpretation involves consideration of whether the apparent increases in disease occurrence, within a specific population at a particular time and place, represent true increases. Possibilities for variation include an increase in population size, improvement in diagnostic procedures, enhanced reporting, duplicate reporting, and other changes in the system [123]. Understanding of the sources of possible study biases can help interpretation of results [124, 125]. In many instances it may be difficult to decide if the change detected is real or artificial, but this question must be answered before action can be contemplated [31]. 

#### 3.2.4. Information Products/Dissemination

The final stage in the public health surveillance process—the timely communication of information to users—is important for follow-up action [77]. Users of surveillance are those who need to know for program planning and decision-making purposes [123]. They include public health practitioners, health planners, epidemiologists, researchers, and policy-makers [77] as well as members of the public and the media [31]. In addition, recipients should include those who provide reports and those who collect the data [123].

Public health decision making depends on three types of knowledge (surveillance, scientific research, and lay experience) [77]. Surveillance knowledge includes statistics that measure health outcomes, health care performance, and other determinants of health. Public health practitioners need to understand and effectively communicate these three complementary types of knowledge. This may involve the use of a variety of communication vehicles such as formal surveillance reports or bulletins, annual reports, teleconferences with partners, media conferences, media releases, and public advisories [77].

While control efforts are not normally seen as a part of surveillance, the link to public health practice is essential. The reason for collecting, analyzing, and disseminating information on a disease is to control that disease. It has been suggested that “*collection and analysis should not be allowed to consume resources if action does not follow*” [126].

#### 3.2.5. Evaluation of Surveillance Systems

Every surveillance system should be evaluated periodically to ensure that it is serving a useful public health function and is meeting its objectives [127, 128]. Several guidelines have been proposed for the evaluation of surveillance systems [75, 127, 129–131].

A systematic evaluation should address the following six aspects: (1) importance [132], (2) objectives and components [132], (3) usefulness [127], (4) cost [133, 134], (5) data quality (accuracy, representativeness and completeness) [127], and (6) quality of surveillance system (simplicity [128], flexibility [127], portability [127], stability [127], acceptability [135], sensitivity [136–141], predictive value positive [138, 141], representativeness [138, 142], and timeliness [138]). Data and system architecture of a surveillance system should follow four design principles: automated, real-time, routinely useful, and locally useful [143].

A number of limitations have been identified for the current surveillance systems.

1. The current surveillance practice is unable to address adequately either current or new potential challenges to public health [107].
2. The current approach to public health surveillance is fragmented, as the various systems are not well coordinated [107, 144]. Ongoing public health information systems are not always integrated with public health surveillance and prevention activities. Instead, over time, a collection of independent and poorly coordinated surveillance systems has evolved in response to various needs [107].
3. It is difficult to address a new emerging health problem because surveillance for the specific problem usually does not exist [107]. New health problems are not detected through the collection of routine surveillance data.
4. Existing surveillance systems may not provide timely data [107]. Timeliness has two components: timeliness after the occurrence of the health event and timeliness of access to data.
5. Inadequate funding has been a problem with current systems of surveillance [145].

#### 3.2.6. Evaluation of Public Health Actions and Practice

When surveillance information is used to plan and implement public health practice (policies, programs), the surveillance system should also serve to evaluate the success of the public health practice [79, 80]. The objective of program evaluation is to determine as systematically and objectively as possible the relevance, effectiveness, and impact of programs with respect to their objectives [146]. The basic principles of program evaluation involves information, expectation, and attribution [147]. Step-by-step guides for program evaluation are available [148–150].

#### 3.2.7. Some Current Topics: Surveillance Dichotomies

There are a number of concepts in surveillance that are currently under discussion. These dichotomies are described below.

*Epidemiologic Surveillance versus Public Health Surveillance.* In 1965, the World Health Organization established the epidemiologic surveillance unit in the Division of Communicable Diseases [36]. The Division director, Karel Raska, defined surveillance to include “*the epidemiological study of a disease as a dynamic process involving the ecology of the infectious agent, the host, the reservoirs, and the vectors, as well as the complex mechanisms concerned in the spread of infection and the extent to which this spread occurs*” [38]. The 1968 World Health Organization definition of surveillance includes “*the use of epidemiologic information*” [69]. 

On the other hand, Thacker and Berkelman in 1988 started using the term “public health surveillance” and gave the following reasons: “the use of the term epidemiologic to modify surveillance is misleading. Epidemiology is a broad discipline that incorporates research and training that is distinct from a public health process that we call surveillance. … For this reason, in this paper, we will not adhere to the current practice of using the term epidemiologic to modify surveillance. We propose that a more appropriate term is public health surveillance, because its use retains the original benefits of the term epidemiologic cited previously and removes some of the confusion surrounding current practice” [30].

*Surveillance versus Research*. According to Thacker and Berkelman “*Surveillance does not encompass research*” and they noted distinctions between public health surveillance and epidemiologic research [30]. Reasons why surveillance is not research include “*Public health surveillance is essentially descriptive in nature. It describes the occurrence of injury or disease and its determinants in the population, and leads to public health action. Research, in contrast, is experimental in design, aimed at testing a hypothesis by comparing and contrasting groups. Surveillance data are usually limited in detail and relatively inexpensive to obtain,… Research data are often quite complex and detailed and are usually expensive to produce. If we confuse surveillance with research, we may be motivated to collect large amounts of detailed data on each case. The burden of this approach is too great for the resources available for surveillance systems and usually leads to failure*” [98]. 

On the other hand, others pointed out that there are biases and problems in the current sample-based research, what Graunt's work published in 1662 was population-based research, and hope that in the future, population-based research may once again become possible through epidemiologic surveillance. “*Graunt's approach for the analysis of … Bills of Mortality … is consistent with the modern technique of population-based epidemiologic surveillance. In the subsequent 300 years, however, the focus of health research shifted to sample-based studies: cross-sectional, cohort and case-control studies, and clinical trials. It appears that epidemiologic surveillance may come back full circle in the 21st century and become once again the focus of health research*” [74]. With the advance in information technology, it becomes possible that “*health research can be conducted in the next century using well-maintained and well-validated surveillance databases*” [74].

*Surveillance Ending with Information Dissemination versus Surveillance Ending with Public Health Action.* Langmuir in 1963 advocated limiting the use of the term surveillance to the collection, analysis, and dissemination of data. His construct of surveillance ended with “*dissemination of (health outcome-specific) data to those who need to know*” [67] and did not encompass direct responsibility for control activities [36, 56]. Others also felt that although data are important for informing policy making, they may not lead immediately to action [73]. Surveillance, *per se*, does not include the public health actions resulting from the interpretation of the data, as few would envisage the inherent responsibility of surveillance practitioners (i.e., those public health officials responsible for interpreting the data collected) for prevention and control actions [151].

On the other hand, Raska in 1965 defined surveillance much more broadly than Langmuir [38]. In the case of malaria, Raska saw surveillance as encompassing control and prevention activities [36]. Indeed, the WHO definition of malaria surveillance included not only case detection, but also the obtaining of blood films, drug treatment, epidemiologic investigation, and followup [152]. Former CDC director William Foege also felt an essential relationship between information and action: “*The reason for collecting, analyzing, and disseminating information on a disease is to control that disease. Collection and analysis should not be allowed to consume resources if action does not follow*” [126, 153].

*Surveillance versus Monitoring.* “*Surveillance is the routine tracking of disease (disease surveillance) or, less commonly, risk behaviour (behavioural surveillance) using the same data collection system over time*” [154]. Surveillance helps describe an epidemic and its spread and can contribute to predicting future trends and developing prevention programmes. In other words, surveillance is the routine tracking before (or without) an intervention (policy, program, or action), which can lead to the development of an intervention. 

On the other hand, “*monitoring is the routine tracking of priority information about a program and its intended outcomes*” [154]. Monitoring helps determine which areas are in need of greater effort and flags questions which might contribute to an improved response but that can only be answered by more refined outcome research methods than those used in routine surveillance and monitoring. In other words, monitoring is the routine tracking after an intervention is implemented and can lead to the improvement of the intervention.

*Surveillance versus Evaluation.* “*Evaluation is a collection of activities designed to determine the value or worth of a specific programme, intervention or project*” [154]. In other words, it is the determination of the relevance, effectiveness and impact of a program with respect to its objectives [146]. It involves three steps: information, expectation, and attribution [147]. Evaluation uses the same methods as in surveillance to collect information. But it goes two steps further than surveillance. It compares the actual program impacts to the expected level as specified by the program objectives (expectation). It also tries to attribute the changes to the development and implementation of the program (attribution).

There is a difference between a program's monitoring and evaluation [154]. Monitoring tracks changes in outcomes following the implementation of a program or project but is not able to attribute those changes directly to the intervention. Evaluation is designed specifically to be able to attribute the changes to the intervention itself and not the result of nonprogram factors.

When a surveillance system (as a public health action) is monitored or evaluated, that means a second surveillance system is created on the surveillance system itself [155]. While the surveillance system uncovers problems in the general population, the second surveillance system uncovers problems in the surveillance system. In other words, the evaluation of surveillance is “*surveillance of surveillance*” [156]. For monitoring or evaluation of policies, the aim is to “*police the policies*” [156].

*Passive Surveillance versus Active Surveillance.* Passive surveillance systems refer to routine notifiable-disease reporting [134]. This is simple and not burdensome to the health department but is limited by incompleteness in reporting [99]. Also, because passive surveillance depends on people in different institutions to provide data, data quality and timeliness are difficult to control [102]. 

To overcome limitations of passive systems, active surveillance systems involve regular outreach to potential reporters to stimulate the reporting of specific diseases [134]. This can be used to validate the representativeness and completeness of passive reporting [99]. As active surveillance employs staff members to regularly contact heath care providers or the population to seek information about health conditions, it provides the most accurate and timely information, but it is also expensive [102].

*Chronic Disease Surveillance versus Communicable Disease Surveillance.* There are differences between chronic and communicable disease surveillance methodologies, in terms of temporality, disease course, cause of disease, public health intervention, data sources, data collection, legislation and regulations, and comorbidity [157].

*Surveillance System versus Health Information System.* Surveillance systems have the capacity to collect, analyze, and disseminate data to public health programs and regularly evaluate the effectiveness of the disseminated data [30, 67, 158, 159]. 

On the other hand, health information systems include a variety of data sources essential to public health and are often used for surveillance; however, they lack some critical elements of surveillance systems. For example, they may not focus on specific outcomes (e.g., vital statistics), are not ongoing (e.g., a onetime or occasional survey), or are not linked directly to public health practices (e.g., insurance claims data) [107]. Health information systems encompass all the different data collection systems available to a ministry of health, including information from hospitals, clinics, and providers (such as the numbers of patients, diagnoses, procedures, and outcomes; personnel, and pharmaceutical and other procurement systems; program-specific data such as vaccinations, prenatal care, and disease treatment outcomes) [98]. However, it has been suggested that “*public health surveillance is one component of the health information system*” [98].

*Vertical Surveillance versus Integrated Surveillance.* Vertical surveillance systems focus on one disease or injury. Information is then fed back into the specific disease control program [98]. 

On the other hand, an integrated surveillance system envisages a common system for multiple diseases using similar structure, processes, and personnel. This requires coordination but is more efficient and less costly, because it allows building on existing resources and capacity. It also promotes the most effective use of health resources [98].

*Universal Surveillance versus Sentinel Surveillance.* Universal surveillance attempts to gather surveillance data from all reporting sources (e.g., hospitals, agencies, health care workers). Universal surveillance is preferred for chronic diseases, where all cases can be ascertained through routine administrative databases, such as hospital records.

On the other hand, sentinel surveillance selects, either randomly or intentionally, a small group of reporting sources who agree to report all cases of one or more notifiable conditions. These designated reporting sources then receive greater attention from health authorities than would be possible with universal surveillance [160]. Sentinel surveillance may require more time and resources, but can often produce more detailed data on cases of illness. For example, sentinel influenza surveillance can collect nasopharyngeal swabs from each patient at selected sites to identify the type of influenza virus, but collection of such data from all patients would not be possible [160]. Others propose that notification of all cases is only necessary for the very limited group of diseases which are rare or for which case finding may be necessary. Information for epidemiological purposes is required for a very wide range of relatively common infectious diseases and could best be obtained from a small number of sentinels [137]. This would give a more accurate picture of a sample of the population, from which extrapolation for national and international comparisons could be done [161].

## 4. The Future

The future cannot be predicted with certainty. This section on the future of surveillance is based on a review of articles that have presented views on the future. Also some current efforts and activities that may affect future directions are also summarized below.

### 4.1. Exploring New Frontiers for Public Health Surveillance

Historically, surveillance focused on infectious disease, then broadened to other topics, including chronic diseases, such as cancer, then diabetes [162]. The 1980s and 1990s also saw surveillance concepts applied to such new areas of public health as occupational health [163, 164], environmental health [101, 165], hazard surveillance (toxic chemicals and physical and biological agents) [166], emerging infectious diseases [167], injury control [168, 169], behavioural risk factors [170], events following disasters [171], pharmacosurveillance [172], and firearm-related injury [173]. At this time, mental health and mental illness are also recognized as domains in public health surveillance [76]. It is expected that further new frontiers will be explored in the future for surveillance.

New frontiers mean new challenges and solutions. Let us take mental health and mental illness as an example. According to the World Health Organization, mental illnesses account for more collective disability burden in developed countries than any other group of illnesses, such as cancer and heart disease [174]. Although mental health measures are now included in established health surveys, there are challenges not seen before. The ways different surveys define and measure mental illnesses often vary and are based on different approaches (such as symptoms, duration, frequency, reference periods, mental health measures, and method of data collection) [175]. In discussing future directions for public health surveillance, Freeman and colleagues suggest that *“(F)uture public health surveillance systems should incorporate measures of positive psychological function as both a protective factor against poor health outcomes and a mental health indicator of interest in its own right*” [76]. Surveillance has traditionally focused largely on established disease or symptoms, but collection of additional data on resilience, coping skills, protective factors, and aspects of positive mental health are considerations in devising strategies for disease prevention and mental health promotion. Maintaining focus on the overall health of our population will be critical in the next decades, as will leaving behind the commonly accepted divide between mental and physical illnesses, “*despite the fact that both exist within individuals in an exquisitely integrated fashion*” [176]. In the future, an optimal surveillance system will examine interactions among biological, social, psychological, and environmental factors to support health promotion, intervention programs, and both mental illness and chronic disease prevention.

### 4.2. Enhancing the Use of Computer Technology in Public Health Surveillance

Use of computer technology, although not without problems [177], continues to contribute to the evolution of public health surveillance [36, 178]. For example, by 1991 in the United States, the National Electronic Telecommunications Systems for Surveillance (NETSS) had linked all state health departments in the country by computer for the routine collection, analysis, and dissemination of information on notifiable conditions [179]. In 2001, the US CDC began implementing the National Electronic Disease Surveillance System (NEDSS) to better manage and enhance the large number of current surveillance systems and allow the public health community to respond more quickly to public health threats (e.g., outbreaks of emerging infectious diseases and bioterrorism) [75]. In 2007, 35 US states had integrated public health surveillance systems as articulated in the NEDSS vision [180]. When NEDSS is fully implemented across the United States, public health professionals and government agencies will be able to quickly recognize and respond in real-time to disease outbreaks or bioterrorism attacks.The Minitel system used in France has also demonstrated the essential utility of office-based surveillance for a variety of conditions of public health importance [36, 181].

Public health surveillance relies on public health information systems that have been defined to include a variety of data sources essential to public health action [107]. Computer technology can improve these public health information systems which vary from a simple system collecting data from a single source, to electronic systems that receive data from many sources in multiple formats, to complex surveys. As the number and variety of systems will likely increase, future efforts of public health surveillance should focus on advances in electronic data interchange and integration of data, which will also heighten the importance of patient privacy, data confidentiality, and system security [107].

There is great interest over the potential that new computer technology will improve the quality, capacity, and effectiveness of public health surveillance systems. One example is the use of a promising interactive health information technology called “eHealth” [182]. eHealth (also written e-health) is a relatively recent term for healthcare practice supported by electronic processes and communication [183]. Other technologies include a novel approach that was presented for detecting influenza outbreaks using search engine query data [184]. Historical logs of more than 50 million of the most common online Web search queries in the United States were analyzed to track influenza-like illness in different areas and regions of the country. There was a high correlation of Google queries (influenza-like illness-related search queries) with the percentage of physician visits in patients with influenza-like symptoms. Another example is a recent analysis of how Internet surveillance tools can assist in the early identification of disease outbreaks [185]. The study found that Web-based sources of information allow timely detection of outbreaks, reduce cost, increase reporting transparency, and presented a list of major advantages and disadvantages of “Internet-based surveillance.”

New terms like “infodemiology” and “infoveillance” have been coined for the use of informatics methods to analyze queries from the Internet search engines to predict disease outbreaks [186]. Public health informatics is “*the systematic application of information and computer science and technology to public health practice, research, and learning*” [187]. Public health informatics can introduce new applications to broaden public health perspectives, strengthen prevention in public health, and build healthier communities [188, 189].

### 4.3. Improving Methods of Epidemic Investigations

New science and technology will continue to improve the approach to epidemiologic outbreak investigations. Rapid technology development in the laboratory has improved diagnostic precision and reduced the time necessary to make a diagnosis. These improvements should continue, for example, to identify pathogens in imported foods at the place of importation and among persons who now travel more extensively and more rapidly around the globe [190]. Similarly, increased use of electronic health records (one form of eHealth [182]) will facilitate more timely and accurate data collection as well as real-time dissemination of recommended control measures to clinicians and health-care facilities. Statisticians continue to develop new statistical methods that will provide insights through refined data analysis. For example, mathematical modeling, especially in complex and time-consuming investigations (e.g., pandemic influenza), can enable application of control measures to reduce the number of cases that are epidemic related. Improved techniques for training also need to be developed so that the technology of epidemic investigations can be used effectively by public health practitioners [190].

### 4.4. Improving Methods of Data Collection

Telephone surveys have been a powerful tool for data collection. However, the use of telephone-based random-digit-dialling methods in public health surveys and surveillance is now at a crossroads [191]. Rapid changes in telecommunication, declines in participation rates, increases in the required level of effort and associated costs are becoming key challenges for telephone surveys. 

It will be important to continue to improve existing methodology and develop new cost-effective and valid data collection methodologies for the future. There has been recent success. Split-sample experiments conducted to examine the impact on survey participation rates of sending advance letters and leaving scripted messages on the answering machines of potential sample members found that whereas advance letters significantly increased response rates, decreased initial refusal rates, and increased refusal conversion rates, leaving messages on telephone answering machines was not an effective strategy [192, 193]. A time-series study found that the “Do Not Call Registry” available in many countries has no significant impact on survey response rates [194]. Real-time interpretation during a telephone survey, which can expand the number of languages in which surveys are offered, was found to produce favourable results [195]. Other studies examined using mixed survey modes, such as internet and mail questionnaires, to increase participation in telephone surveys [196, 197]. Recent survey methodology studies also found address-based sampling to be promising [198, 199] and cellular telephone surveys to be able to reach the young age groups that were most likely to be excluded by random digit dialling [200, 201]. The American Association for Public Opinion Research 2012 Conference discussed new frontiers in public opinion research, including cell phone surveys, surveys with proxies and with immigrants, dual frame sample, mixed mode survey, optimal sample allocation, interactive and gaming techniques, and linking data with action [202].

Future surveys should collect physical measurements as part of its ongoing operation, as several studies have examined the limitations of self-reported survey data and their impact on estimates such as obesity and blood pressure using national surveys [203, 204]. 

In future, standards need to be developed that are common to all datasets as well as unique to individual datasets. Examples include minimum lists of demographic variables and ICD codes, standardized codes for demographic variables, a minimum set of statistical tests, common definitions of statistical tests, and rules for minimum cell size suppression. Examples of standards unique to individual datasets include rules governing the development of life tables from mortality datasets and the development of fertility rates from birth files [205]. 

A number of methodological research areas to improve data collection in the 21st century have been suggested such as systematic process for indicator selection; methodology to convert results from different health surveys with different indicator definitions to a standard and compatible level; methodology to increase survey response rates by population subgroups; methodology to collect proxy indicators; incorporation of laboratory data in routine population health surveillance; development of automatic, laboratory-based, and electronic reporting of diseases [74].

### 4.5. Improving Methods of Epidemiologic and Statistical Analysis

A renewed activity associated with public health surveillance is that of the methods of epidemiologic and statistical analysis [36]. Since the 1980s, applications and methods of time series methods have enabled more meaningful analysis and interpretation of data collected from surveillance [206]. More sophisticated techniques such as geographical and spatial methods and space-time monitoring will continue to be applied to public health surveillance as they are developed [207].

Postsurvey adjustments are becoming an increasingly important means of maintaining the representativeness of survey data. New weighting methods have been developed for adjusting the data for sex, age, race, education, marital status, and telephone coverage [208], and for nontelephone coverage [209].

A number of methodological research areas to improve data analysis in the 21st century include application of “capture-recapture” methodology to identify missing cases in routine data; conditions in which age-standardized techniques can be used for time trend and geographic comparisons; development of economic analysis models; methodology for multilevel analyses [74].

### 4.6. Improving Methods of Information Dissemination

Since the latter part of the 19th century, the dissemination of surveillance information generally has been done by “weekly reports” of diseases of critical health or strategic importance [210]. In the United States, the Weekly Abstract of Sanitary Reports [211], published since 1886, has included morbidity and mortality information for most cities and ports of the United States and many countries of the world [210]. Until recently, surveillance information was disseminated as written documents published periodically by government agencies [36]. 

Although printed paper reports will continue to be produced, there is a need to explore new methods of information dissemination, such as paperless or electronic media [212]. Associated with ready electronic access to detailed personal information from surveillance are ethical and legal concerns that might constrain access to data of potential public health importance [213].

A number of methodological research areas to improve information dissemination in the 21st century are methodology to alert health professionals and the general public about forthcoming health risks; innovative and nontraditional methods for information dissemination; methods to put our current knowledge of risk assessment and management into perspective so the general public knows what health risks to avoid (e.g., publication of “Handbook of Health Risks”) and what healthy activities to pursue (e.g., publication of “Handbook of Healthy Practices”); ongoing and timely information dissemination system; survey of the general public for their regular and most effective channels of obtaining health information; development of summary indicators for health, risk and intervention (e.g., for Canada, Canadian Health Index, Canadian Heart Health Index, Canadian Diet Index) in a way similar to the Consumer Price Index or stock market indices; development of 365 health, risk, and intervention indicators for reporting to the general public after the evening television news, one indicator a day; computer software to calculate probability of risks of selected diseases or overall health outcomes, based on input concerning personal lifestyle, demographics, diet, and smoking (e.g., as “hands-on” project to be placed in science museums) [74].

### 4.7. Improving Use of Surveillance Information by Decision Makers

Perhaps most importantly, surveillance information should be used more by decision makers [4, 36]. It must, however, be recognized that while public health surveillance is the cornerstone of public health practice [214], it is not the only source of information for evidence-based public health [77], as surveillance is only one element in the package of evidence to influence healthy public policies [4]. There are at least 5 tools/processes for decision makers on public health actions: meta-analysis, risk assessment, economic evaluation, public health surveillance, and expert panels/consensus conferences [215]. In addition to scientific evidence, policy making is also based on values, emotions, and the wishes of interest groups [216]. 

Because public health surveillance and action are crucial to effective public health practice, the World Health Organization has initiated consensus meetings at the regional and national level to review and reform surveillance and action systems [217–220]. These meetings emphasized improved epidemic preparedness and epidemic response. They also highlight the need to facilitate and standardize surveillance and action assessments and to include integration strategies in the reform process.

In 1992, “The Nation's Health Report Card”, published by the American Public Health Association, provides state-by-state rates of health status, health risk, health determinants, and health service indicators [221]. In the future, the concept of a national health report card can be extended to “community health report cards”, using data on residents of an entire community [222, 223].

### 4.8. Building the Future Based on Lessons Learned from the Past

Summarizing the lessons learned from a review of historical perspective of epidemics from the past five millennia, Choi and Pak proposed 12 future challenges for public health surveillance in the 21st century to (1) expand the current surveillance system to include besides deaths also new cases for diseases; (2) develop long-term plans for surveillance systems and to avoid ad hoc systems; (3) develop ground rules on when and how to add or delete or change the definitions of variables under surveillance when new scientific evidence arises; (4) develop large scale and widespread data collection systems which are population-based (see also [224]); (5) expand the current surveillance system based mainly on health outcomes to also include risk factors and intervention indicators; (6) develop novel analysis tools and new statistics to facilitate development of disease prevention and control strategies; (7) develop surveillance systems that are closely integrated with etiologic research; (8) develop better and more accurate methods for forecasting; (9) develop a more direct and effective mechanism to feed information into the public health decision making process; (10) develop better evaluation protocols for public health programs and intervention using surveillance data; (11) develop better ways of dissemination of information to all those who need to know; (12) ensure that the surveillance system would achieve health for all, on an equal basis and without prejudice [7].

### 4.9. Building Surveillance Capacity

To avoid fragmentation in national surveillance efforts [107, 144], there is a need for federal agencies to provide national facilitation to foster interstate and intercounty collaboration. Central guidance can lead to coordination across states and counties, interstate technology transfer, and opportunity to learn from the successes and failures of other localities. Needless expense, unnecessary development time, and failure to rapidly share information on innovative systems can be avoided [205]. No attempt to meet the current challenges in public health surveillance will succeed unless it recognizes the fundamental importance of providing and maintaining a cadre of highly trained and motivated public health professionals in every local health agency in the country [5]. To use surveillance information to better prioritize, plan, deliver, and evaluate programming, public health staff must possess the required knowledge and skills. While it is neither feasible nor necessary for all staff to receive postgraduate academic training, a greater proportion of the public health workforce will need to acquire the knowledge and skills necessary to effectively understand and use surveillance concepts and techniques. Public health surveillance systems must be strengthened by (1) allocating resources, including human resources, for the effective use of health surveillance data and tools and (2) recognizing the need for existing staff to acquire new skills [77].

There are different challenges in building public health workforce capacity in developed countries [223, 225] and developing countries [226, 227].

### 4.10. Enhancing Global Public Health Surveillance

Globalization of trade and the economy has resulted in a constant massive mobilization of commodities and people across countries and continents at unprecedented speed. It takes only a few hours to transport or mobilize thousands of people and goods across the globe. It is possible to travel between most places in the world in less time than the incubation period for many infectious diseases [228]. There is also a need for global surveillance for risk factors for chronic diseases, as risk factors are transferable. International migrants bring with them their cooking styles, hygiene practices, and so forth, thereby affecting both the infectious and chronic disease patterns in the host country [229]. In this sense, chronic noncommunicable diseases like cardiovascular diseases can be considered communicable [230].

Three directions of global surveillance are transforming the functions of public health in a globalized world: (1) the role of the new International Health Regulations (IHR) [231, 232], (2) the emergence of new global health surveillance networks, and (3) the reshaping of guidelines for the collection, dissemination, and interventions in global surveillance [210]. The revised IHR of 2005 encourage a new paradigm of global public health intelligence [233]. With mandatory reporting procedures and requirements for building surveillance and response capacity, the revised IHR are a move toward more effective global health security [234].

In 1997, the “Global Public Health Intelligence Network” was proposed by the World Health Organization in partnership with the Public Health Agency of Canada to help identify significant disease outbreaks around the world taking advantage of the existing globalized virtual communications [235]. This global surveillance initiative is an Internet surveillance system that gathers data and public health reports from diverse countries in 7 languages, aiming to disseminate timely alerts to help control outbreaks, the spread of infectious disease, contamination of food and water, bioterrorism, natural disasters, and exposure to chemical agents and nuclear materials. This system investigates and confirms outbreak reports of global health significance [236] and also monitors questions related to the safety of medications and medical products [237].

The World Health Organization has created a global network of national influenza centres in 83 countries [236], the “FluNet” [238] and “DengueNet” [239], as Internet sites dedicated to monitoring global influenza and dengue-related information. A network of Internet-based surveillance, “ProMED-mail”, initiated by the International Society of Infectious Diseases, is considered to be one of the largest publicly available Internet-based reporting networks for emerging diseases in the world [240, 241].

In global chronic disease surveillance, new global health surveillance networks have also emerged. Examples include the World Alliance for Risk Factor Surveillance (WARFS) [242] and the Americas' Network for Chronic Disease Surveillance (AMNET) [243]. WARFS is the Global Working Group on Surveillance of the International Union for Health Promotion and Education (IUHPE). It supports the development of behavioural risk factor surveillance as a tool for evidence-based public health, acknowledging the importance of this information source to inform, monitor, and evaluate disease prevention and health promotion policies, services, and interventions. There has been a series of biennial global conferences on risk factor surveillance, beginning in USA (Atlanta), 1999; Finland (Tuusula), 2001; Australia (Noosaville), 2003; Uruguay (Montevideo), 2005; Italy (Rome), 2007; Italy (Venice), 2009; Canada (Toronto), 2011. AMNET was established in Uruguay in 2003 as a regional network for the purposes of sharing information and experiences as well as providing opportunities for enhancing chronic disease surveillance in the WHO Region of Americas (North, Central and South America, and the Caribbean) [156].

Global health is seen in several developed countries as a pillar of their foreign policy [244, 245]. Several governments, including US [246], Canada [247], and UK [248], are expanding their investment in global health and global security.

## 5. Conclusion

Emerging infectious diseases, such as human immunodeficiency virus/acquired immunodeficiency syndrome (HIV/AIDS), severe acute respiratory syndrome (SARS), and pandemic influenza, and emerging chronic conditions, such as the global obesity epidemic, have demonstrated that we remain vulnerable to health threats [249]. The importance of strengthening public health surveillance to provide early warning and develop actions has been a primary focus in public health. However, despite improvements in the past decades, public health surveillance capabilities remain limited and fragmented, with uneven global coverage. 

It is hoped that learning from the past, reflecting on the present, and planning for the future can further enhance public health surveillance for the good of humankind.

## Figures and Tables

**Table 1:** Some major epidemics in human history.

Year*	Place	Event
3180 B.C.	Egypt	*First recorded epidemic*: “A great pestilence” during the reign of Pharaoh Mempses in the First Dynasty was the first recorded epidemic in human history [7, 9].

1495 B.C.	Egypt	“The Plague of Pharaoh”, possibly caused by drought [7, 10].

1471 B.C.	Kadesh	A plague causing 14700 deaths, possibly caused by earthquake [7, 9].

1190 B.C.	Greece	A *loimos* (Greek, meaning a plague or pestilence), now believed to be a bubonic plague, possibly caused by the Trojan War (1194–1184 B.C.) [7, 9].

1017 B.C.	Israel	A pestilence lasing “3 days”, causing 70000 deaths [7, 9].

431 B.C.– 427 B.C.	Aethiopia, then spread to Egypt, the Persian Empire, and Athens	“The Plaque of Thucydides”, now believed to be typhus and measles, possibly caused by the Peloponnesian War (432–411 B.C.) [7, 9].

A.D. 166	Rome	Possibly smallpox, spread by soldiers returning from the Parthian War (A.D. 161–166) [7, 11].

541–549	Constantinople, then spread to Egypt and the whole populated world	*First of three most devastating epidemics to hit the human race* [9]: “The Plague of Justinian” [7, 9].

664–689	England	“The Yellow Plague”, now believed to be relapsing fever with jaundice, causing death of “a great multitude of men” [7, 9].

1348–1351	Central Asia, then spread east to China, south to India, west to Portugal, north to England (1349), Norway (1350), and Russia (1351)	*Second of three most devastating epidemics to hit the human race* [9]: “The Black Death”, now believed to be bubonic plague, with “thousands died everyday”, possibly caused by contaminated ships following the trade routes [7, 9]. Quarantine was used to detain travelers from infected areas [12].

1374–17th century	Germany (1374), then spread to France (1518) and Italy (17th century)	“Dancing Mania”, possibly caused by mass psychogenic disorder and/or the bite of a spider [7, 9].

1665	London	“The Great Plague of London”, caused by poor sanitary conditions, dense population, and overcrowded housing. The epidemic was ended by natural interventions, with winter frosts, and the “Great Fire of London” in 1666 that destroyed and cleansed the neighbourhoods [7, 9].

1817–1875	Calcutta (1817), all of India (1821), China (1820), Japan (1822), Russia (1823), England (1831), Canada and the USA (1832), Africa (1837), Central America (1863), and South America (1875)	Four pandemics of cholera (1817–1823; 1826–1837; 1846–1863; 1863–1875), caused by steamboats and mass migration during the Industrial Revolution [7, 9]. In 1849, John Snow mapped cholera cases in London and found contaminated water from the Broad Street pump. Snow removed the pump handle in 1854 and the epidemic waned [13].

1918	France (April), England (June), China (July), and USA (August)	*Third of three most devastating epidemics to hit the human race* [9]: “Spanish Influenza”, caused by a virus [7, 14]. The disease killed 22 million people, about twice as many as the 10 million deaths caused by World War I (1914–1918) [14]. The virus was isolated in 1933, and its vaccine was developed in 1972 [9]. Facemasks and hand washing have been suggested for preventing the spread of influenza [15].

1940–now	Worldwide	Lung cancer epidemic, caused by cigarette smoking [7, 16, 17]. In the decade 1990–1999, a total of 6.6 million attributable deaths worldwide [18, 19].

1997–now	Worldwide	Obesity epidemic, caused by a combination of excess food intake, lack of physical activity, and genetic susceptibility [20, 21]. Before the 20th century, obesity was rare [22]. In 1997 the World Health Organization formally recognized obesity as a global epidemic [23]. World Health Organization estimates that 1.4 billion adults are overweight or obese, and 2.8 million adults die each year as a result of being overweight or obese [24].

*Year refers to the time when an epidemic was first reported in a place. The epidemic could recur in a place subsequent to the year cited.

**Table 2:** Three types of information in the historical records of epidemics (based on an analysis of Table 1).

Health outcomes	Risk factors	Interventions
Plague	Drought	Quarantine
Smallpox	Earthquake	Winter frosts
Relapsing fever	War	Great Fire of London (1666),
Jaundice	Bacteria	Good sanitation
Dancing Mania	Psychological	Broad street pump (1875),
Cholera	Spider	Hand washing
Influenza	Poor sanitation	Vaccine
Lung cancer	Overcrowding,	No smoking
Obesity	Virus	Smoking cessation
	Tobacco	Healthy food
	Excess food	Physical activity
	Physical inactivity	

**Table 3:** Some major milestones in the historical development of public health surveillance.

Year	Place	Event
3180 B.C.	Egypt	*First recorded epidemic*: “A great pestilence” [7, 9].

460 B.C.–370 B.C.	Greece	*Father of medicine*: Hippocrates wrote about the endemic state and epidemic state of disease [25].

1348	Venice	*First public health action that can be attributed to surveillance*: during the “Black Death”, three guardians of public health for the Republic of Venice prohibited ships with infected passengers from docking at the port [26, 27].

1532	London	*First systematic ongoing collection of surveillance data*: England started collecting the London Bills of Mortality [28].

1662	London	*First comprehensive analysis and interpretation of mortality data*: John Graunt, based on an analysis of the Bills of Mortality, published the “Natural and political observations made upon the bills of mortality” [29].

1665	London	*First epidemic field investigation*: during the “Great Plague of London”, the diarist Samuel Pepys recorded the weekly number of deaths and made observations on the extent and progression of the epidemic [7].

1741	Rhode Island	*First legislation for surveillance*: the American colony of Rhode Island required by law that tavern-keepers report contagious disease among their patrons [30].

1766	Germany	*First link of surveillance to policy*: Johann Peter Frank encouraged linking surveillance to public health policy, such as school health and public water and sewage treatment [30].

1788–1799	France	*First declaration that public health is the responsibility of the state*: leaders of the French Revolution declared health of the people to be the responsibility of the state [27].

1834	England	*First link of surveillance to legislation*: Sir Edwin Chadwick used surveillance data to demonstrate the link between poverty and disease [31]. This led to the Poor Law Amendment Act 1834.

1838	England	*Founder of the modern concept of surveillance*: William Farr was appointed as the first Compiler of Abstract (i.e. medical statistician) and created a surveillance system that has earned him recognition as the founder of the modern concept of surveillance [25, 32].

1850	United States	*First link of surveillance to statewide public health infrastructure*: Lemuel Shattuck published a report based on a survey of sanitary conditions in Massachusetts and recommended a census and collection of health data [31].

1854	London	*Father of modern epidemiology*: John Snow is widely regarded as the father of modern epidemiology for his work in 1854 in tracing a deadly cholera outbreak to a contaminated water pump on Broad Street [33].

1874	United States	*First systematic reporting of infectious diseases*: Massachusetts State Board of Health instituted a plan for physicians to provide weekly reports on prevalent diseases, using a standard postcard-reporting format [34, 35].

1888	Italy	Mandatory reporting of eleven communicable diseases and death certificates [27].

1890	United Kingdom	Compulsory reporting of infectious diseases [36].

1893	United Kingdom	Publication of international list of causes of death by the International Statistical Institute (founded in London in 1885) [27].

1911	United Kingdom	Use of National Health Insurance data for surveillance [27].

1925	USA	All states participated in national morbidity reporting after the severe poliomyelitis epidemic of 1916 and influenza pandemic of 1918-1919 [37].

1935	USA	First national health survey [27, 36].

1943	Denmark	First registry, the Danish Cancer Registry [27].

1943	United Kingdom	First Sickness Survey [27].

1965	Geneva	Establishment of an Epidemiological Surveillance Unit in the Division of Communicable Diseases at World Health Organization headquarters [38].

1966	Geneva	First publication of Communicable Disease Surveillance Reports by World Health Organization [27].

1967	United Kingdom and the Netherlands	Development of General Practitioners' Sentinel Systems [27].

1968	Geneva	The 21st World Health Assembly established surveillance as an essential function of public health practice [39].

**Table 4:** The first epidemic field investigation based on the diary of Samuel Pepys during the Great Plague of London, 1665 [49].

Date	Deaths from plague	Total deaths	Proportionate mortality
June 8	43		
June 15	112		
July 20	1089		
Aug 31	6102	7496	81%
Nov 30	333	544	61%

**Table 5:** The historical evolvement of the definitions of public health surveillance.

Year	Author	Definition	Remarks
1662	John Graunt	“*Now having engaged my thoughts upon the Bills of Mortality, and so far succeeded therein, as to have reduced several great confused Volumes into a few perspicuous Tables, and abridged such Observations as naturally flowed from them, into a few succinct Paragraphs … I hoped … to see unto how much profit that one Talent might be improved, beside the many curiosities concerning the waxing and waning of Diseases*” [29].	Surveillance is based upon successful analysis of population-based, on-going data (e.g., death records). There are several basic principles of data analysis: reduce volumes of data to a few easy-to-understand tables, then interpret them, and prepare a few brief and precise paragraphs, so as to gain profit from the data analysis, in order to understand the increase and decrease of diseases [7].
1687	Sir William Petty	“*Political Arithmetic*” [66].	“*Much of the data manipulation that epidemiologists do requires a fourth grade education in arithmetic. However, the wisdom as to the validity of the data and the conservatism of interpretation requires persons with a keen political sense*” [46].
1963	Alexander Langmuir	“*Surveillance, when applied to a disease, means the continued watchfulness over the distribution and trends of incidence through the systematic collection, consolidation and evaluation of morbidity and mortality reports and other relevant data. Intrinsic in the concept is the regular dissemination of the basic data and interpretations to all who have contributed and to all others who need to know*” [67].	“*Langmuir was careful to distinguish surveillance both from direct responsibility for control activities and from epidemiologic research, although he recognized the important interplay among epidemiologic studies, surveillance, and control activities*” [30]. “*Langmuir stated on more than one occasion that the concept of surveillance did not encompass direct responsibility for control activities*” [27], and that “*the surveillance officer should be the alert eyes and ears of the health officer and he should advise regarding control measures needed, but the decision and the performance of the actual control operations must remain with the properly constituted health authority*" [68].
1968	World Health Organization	Surveillance is the “*systematic collection and use of epidemiologic information for the planning, implementation, and assessment of disease control*” [69].	In the sense of the 1968 definition, surveillance implies “*information for action*” [70].
1986	Centers for Disease Control	“*Epidemiologic surveillance is the ongoing systematic collection, analysis, and interpretation of health data essential to the planning, implementation, and evaluation of public health practice, closely integrated with the timely dissemination of these data to those who need to know. The final link in the surveillance chain is the application of these data to prevention and control*” [71].	“*A critical word in this definition is “ongoing”; one-time surveys or sporadic studies do not constitute surveillance. An ongoing system of data collection and collation is also not sufficient to constitute public health surveillance, because to be useful the data must be integrated into the conduct and evaluation of specific public health programs, which may include epidemiologic research leading to prevention*” [30]. “*The 1986 CDC definition of surveillance reflects Langmuir's 1963 view and avoids the use of the term surveillance for control activities, although it states that the final link in the surveillance chain is the application of these data to prevention and control*” [27]. “*The 1986 CDC concept of surveillance differentiates surveillance from occasional surveys and from planned comprehensive research programs*” [72].
1988	Thacker and Berkelman	“*Public health surveillance is the ongoing systematic collection, analysis, and interpretation of outcome-specific data, closely integrated with the timely dissemination of these data to those responsible for preventing and controlling disease or injury*” [30].	“*This definition, however, contains two very different activities. Case surveillance focuses on individuals, to identify those with certain diseases and take action. Statistical surveillance, on the other hand, focuses on populations, to identify differentials and trends that can inform public health policymaking, including the allocation of resources*” [73].
1998	Bernard Choi	“*A surveillance system is … a systematic, ongoing and population-based system for the collection, analysis, and interpretation of data on health outcomes, risk factors, and intervention strategies, for the monitoring and early warning of health events, and for the development and evaluation of public health interventions and programmes, closely integrated with the timely dissemination of the information to those who need to know*” [7, 74].	Based on 12 lessons learned from the past 5000 years of the history of epidemics, a surveillance system should have twelve desirable features, including (1) evolving, (2) ongoing, (3) systematic, (4) population-based, (5) comprehensive, (6) analytic, (7) hypothesis generating, (8) early warning, (9) informing programs and interventions, (10) evaluative, (11) effective in information dissemination, and (12) equitable [7].
2001	US Centers for Disease Control and Prevention	“*Public health surveillance is the ongoing, systematic collection, analysis, interpretation, and dissemination of data regarding a health-related event for use in public health action to reduce morbidity and mortality and to improve health*” [75].	“*Historically, surveillance focused on infectious disease, then broadened to other topics, including chronic diseases*” [76].
2006	Public Health Agency of Canada, Public Health Research, Education and Development, Canadian Public Health Association	“*Health surveillance is the ongoing, systematic use of routinely collected health data to guide public health action in a timely fashion*” [77].	
2012	World Health Organization	“*Surveillance is systematic ongoing collection, collation and analysis of data and the timely dissemination of information to those who need to know so that action can be taken*” [78].	
2012	World Health Organization	“*Public health surveillance is an ongoing, systematic collection, analysis and interpretation of health-related data essential to the planning, implementation, and evaluation of public health practice*” [79].	
2012	World Health Organization	“*Public health surveillance is the continuous, systematic collection, analysis and interpretation of health-related data needed for the planning, implementation, and evaluation of public health practice*” [80].	

**Table 6:** Comparison of definitions of public health surveillance over time.

Year	1662	1687	1963	1968	1986	1988	1998	2001	2006	2012
	Graunt	Petty	Langmuir	WHO	CDC	Thacker and Berkelman	Choi	CDC	PHAC	WHO
(1) Name										
Natural and political observations	*✓*									
Political arithmetic		*✓*								
Surveillance				*✓*			*✓*			*✓*
Disease surveillance			*✓*							
Health surveillance									*✓*	
Epidemiologic surveillance				*✓*	*✓*		*✓*			
Public health surveillance						*✓*		*✓*		*✓*

(2) Components										
Ongoing			*✓*		*✓*	*✓*	*✓*	*✓*	*✓*	*✓*
Systematic			*✓*	*✓*	*✓*	*✓*	*✓*	*✓*	*✓*	*✓*
Population-based							*✓*			
Data collection	*✓*	*✓*	*✓*	*✓*	*✓*	*✓*	*✓*	*✓*		*✓*
Mortality data	*✓*	*✓*	*✓*							
Morbidity data			*✓*							
Epidemiologic data				*✓*						
Health data					*✓*		*✓*	*✓*	*✓*	*✓*
Other relevant data			*✓*				*✓*			
Data analysis	*✓*	*✓*	*✓*		*✓*	*✓*	*✓*	*✓*		*✓*
Interpretation	*✓*		*✓*		*✓*	*✓*	*✓*	*✓*		*✓*
Dissemination			*✓*		*✓*	*✓*	*✓*	*✓*		*✓*

(3) Purpose										
Curiosities concerning the waxing and waning of diseases	*✓*									
Disease control		*✓*		*✓*	*✓*	*✓*				
Public health practice					*✓*					*✓*
Public health action							*✓*	*✓*	*✓*	*✓*
Planning, implementation, and evaluation of practice				*✓*	*✓*		*✓*			*✓*

Sources of surveillance definitions:

Graunt [29]; Petty [66]; Langmuir [67]; WHO [69]; CDC [71]; Thacker and Berkelman [30]; Choi [74]; CDC [75]; PHAC [77]; WHO [78–80].

**Table 7:** Uses of public health surveillance.

Categories (adapted from the World Bank) [98]	Examples of uses
(1) Early warning: serves as an early warning system to identify new emerging health problems	(i) Recognize cases or clusters of cases to trigger interventions to prevent transmission or reduce morbidity and mortality [98]. (ii) Serve as an early warning system to identify public health emergencies [79]. (iii) Detect epidemics [36, 99].

(2) Impact assessment: assesses public health impacts and trends of new emerging health problems	(i) Assess the public health impact of health events or determine and measure trends [98]. (ii) Estimate magnitude of a health problem [36, 99]. (iii) Document the distribution and spread of a health event [36, 99]. (iv) Portray the natural history of a disease [36, 99]. (v) Understand the economic and health impacts of a public health issue, and the nature and extent to which it disrupts communities [77].

(3) Intervention development and implementation: develops public health interventions and strategies and allocates public health resources	(i) Demonstrate the need for public health intervention programs and resources, and allocate resources during public health planning [98]. (ii) Lead to immediate public health action [75]. (iii) Set priorities and guide public health policy and strategies [79]. (iv) Rapidly communicate information among public health officials and health care workers so they can take appropriate actions to resolve problems [77]. (v) Appropriate and allocate prevention and care resources [36]. (vi) Make informed decisions related to resource allocation [77].

(4) Intervention evaluation: evaluates public health interventions and strategies	(i) Monitor effectiveness of prevention and control measures and intervention strategies [98]. (ii) Evaluate control and prevention measures [36, 99]. (iii) Evaluate programs, policies, and control measures [77]. (iv) Monitor isolation activities [36, 99]. (v) Detect changes in health practice [36]. (vi) Document impact of an intervention or progress towards specified public health targets/goals [79].

(5) Risk assessment: identifies risk factors and high risk populations	(i) Identify high-risk population groups or geographic areas to target interventions and guide analytic studies [98]. (ii) Monitor changes in infectious agents [36, 99]. (iii) Understand the factors that cause health events, both at the individual and community level [77]. (iv) Monitor and clarify the epidemiology of health problems [80]. (v) Reduce the risk of the occurrence of public health crises [77].

(6) Research: supports public health research	(i) Develop hypotheses that lead to analytic studies about risk factors for disease causation, propagation, or progression [98]. (ii) Formulating research hypotheses [75]. (iii) Generate and test hypotheses [36, 99]. (iv) Identify priorities and hypotheses for research [77]. (v) Facilitate epidemiologic and laboratory research [36].
