# Corrigendum to “The Past, Present, and Future of Public Health Surveillance”

**DOI:** 10.1155/2018/6943062

**Published:** 2018-07-02

**Authors:** Bernard C. K. Choi

**Affiliations:** ^1^Injury Prevention Research Centre, Medical College of Shantou University, Shantou 515041, China; ^2^Department of Epidemiology and Community Medicine, University of Ottawa, Ottawa, ON, Canada K1H 8M5

The article titled “The Past, Present, and Future of Public Health Surveillance” [1] was found to contain material from the following published articles. Although they were all cited and some text was quoted, there was inadvertently insufficient use of quotation in the use of these sources, for which the author sincerely apologizes.

Work by other authors:

- Castillo-Salgado [2], which is cited as reference 210.
- Declich and Carter [3], which is cited as reference 27.
- Teutsch and Churchill [4], which is cited as reference 86.
- Freeman et al. [5], which is cited as reference 76.
- Brachman and Thacker [6], which is cited as reference 190.
- McNabb et al. [7], which is cited as reference 151.
- Johnson and Heymann [8], which is cited as reference 33.

Authors' own work:

- Choi [9], which is cited as reference 74.
- Choi and Pak [10], which is cited as reference 7.
